# Neurological and Systemic Manifestations of Melioidosis: A Three-Case Series From Eastern India

**DOI:** 10.7759/cureus.107931

**Published:** 2026-04-28

**Authors:** Sameer K Mehta, Nibedita Mishra, Aparna Prajapati, Saurabh Pathak

**Affiliations:** 1 Medicine, Tata Main Hospital, Jamshedpur, IND; 2 Cardiology, Tata Main Hospital, Jamshedpur, IND; 3 Nephrology, Tata Main Hospital, Jamshedpur, IND

**Keywords:** abscess, burkholderia pseudomallei, carbapenem, emerging infections, india, melioidosis, meningitis, neuromelioidosis

## Abstract

Melioidosis, caused by *Burkholderia pseudomallei*, is an under-recognized infection in India that frequently mimics tuberculosis and pyogenic infections, leading to diagnostic delays. Neurological melioidosis is rare but associated with significant morbidity and mortality. We describe three culture-confirmed cases of melioidosis encountered over a three-month period at a tertiary care center in eastern India. This series is, to our knowledge, the first from eastern India to document a temporally clustered, culture-confirmed spectrum of melioidosis, ranging from systemic bacteremic to fulminant neurological disease, within a single center. A 58-year-old diabetic male presented with fever and gluteal swelling and was diagnosed with systemic melioidosis based on positive blood cultures. He received intravenous meropenem for 14 days, followed by oral trimethoprim-sulfamethoxazole for 12 weeks, in accordance with Darwin guideline recommendations, showing a good clinical response. A 60-year-old diabetic male presented with fever, headache, and seizures, with MRI revealing subdural and epidural collections along with a cortical lesion suggestive of neuromelioidosis. Cerebrospinal fluid (CSF) was obtained and demonstrated neutrophilic pleocytosis with elevated protein. CSF culture did not yield growth. Central nervous system (CNS) involvement is, therefore, designated as probable based on blood-culture confirmation and neuroimaging. He was discharged against medical advice before completing therapy. A 62-year-old dairy farmer developed fulminant meningoencephalitis following environmental exposure and toe trauma. CSF analysis revealed neutrophilic pleocytosis (150 cells/mm³), elevated protein (180 mg/dL), and low glucose (30 mg/dL), consistent with bacterial meningitis. CSF culture was sterile, and blood cultures confirmed *B. pseudomallei*, with CNS involvement classified as probable. He deteriorated despite treatment and was discharged while ventilated. These cases highlight the wide clinical spectrum of melioidosis, the diagnostic challenge posed by neurological involvement, and the importance of early blood culture testing. Radiological findings may closely resemble tuberculosis or pyogenic infections, and hyponatraemia with elevated inflammatory markers may provide supportive clues. Early diagnosis and adherence to prolonged antimicrobial therapy are critical to improving outcomes in this emerging endemic infection.

## Introduction

Melioidosis is a severe and often fatal infection caused by *Burkholderia pseudomallei*, an environmental Gram-negative bacillus present in soil and surface water across tropical regions, with established endemicity in Southeast Asia and northern Australia. Predictive modeling studies indicate that the true global disease burden is substantially underestimated, with India identified as an under-recognized endemic zone [1]. Over the past decade, increasing case detection, from sporadic reports to clustered outbreaks, supports the view that melioidosis is emerging as a significant public health challenge in India [2].

The disease is notorious for its wide clinical range. In the Darwin prospective study involving 540 patients, clinical manifestations included pneumonia, bacteremia, visceral abscesses, septic shock, and chronic suppurative infections [3]. Because melioidosis overlaps clinically and radiologically with tuberculosis and other tropical infections, it is frequently misdiagnosed or diagnosed late [1-4].

Current management requires a two-phase antimicrobial regimen comprising intensive intravenous therapy (10-14 days) followed by prolonged oral eradication therapy (3-6 months) [4]. This extended course reflects the pathogen’s capacity for intracellular persistence and relapse, even after apparent clinical recovery [5].

In India, recent neuromelioidosis outbreaks and genomic analyses reveal heterogeneity of circulating strains and highlight the need for improved diagnostic capacity and awareness among clinicians [6]. Indian epidemiological studies consistently identify diabetes mellitus, chronic renal disease, and environmental exposure as key risk factors [7].

This case series describes three patients diagnosed with melioidosis at a tertiary center in eastern India over a three-month period. To our knowledge, this represents the first documented temporal cluster of culture-confirmed melioidosis from this geographic region, presenting with a spectrum from systemic bacteremic disease to fulminant neurological involvement. The cases emphasize the diagnostic complexities, radiologic mimicry, therapeutic challenges, and systemic barriers that influence patient outcomes in real-world clinical settings.

## Case presentation

Case 1: Systemic bacteremic melioidosis with deep soft-tissue involvement

A 58-year-old male with type 2 diabetes mellitus, hypertension, and a remote history of hepatitis B presented with a four-day history of high-grade fever, chills, loose stools, and a painful swelling over the right gluteal region. His background of diabetes placed him at increased risk for invasive melioidosis [8]. He also reported frequent consumption of raw, unpasteurized milk, suggesting a potential exposure. On examination, he was tachycardic, and the gluteal region showed diffuse induration without fluctuance. Mild abdominal distension and shifting dullness indicated small-volume ascites. Laboratory evaluation demonstrated anemia (hemoglobin: 8.6 g/dL), thrombocytopenia (80,000/µL), hyponatremia (Na⁺: 130 mEq/L), elevated C-reactive protein (CRP), and moderate transaminitis, reflecting severe systemic inflammation. Ultrasound of the abdomen revealed splenomegaly, ascites, and soft-tissue edema. Two blood cultures collected at admission grew *B. pseudomallei*. The patient was initiated on intravenous (IV) meropenem for 14 days, consistent with guideline-directed intensive-phase management for bacteremic melioidosis [4]. This duration (14 days) falls within the Darwin guideline recommendation of 10-14 days of IV therapy for systemic bacteremic disease [4]. He improved significantly, defervesced, and showed normalization of inflammatory markers. He was discharged on oral trimethoprim-sulfamethoxazole (TMP-SMX) for 12 weeks as eradication therapy, consistent with the guideline-recommended range of three to six months, and remained clinically stable at follow-up.

Case 2: Probable neuromelioidosis with subdural and epidural collections

A 60-year-old man with diabetes and hypertension presented with a 10-day history of high-grade fever and severe headache. Initially alert, he later developed focal impaired-awareness seizures during hospitalization. Laboratory evaluation revealed persistent hyponatremia (Na⁺: 128 mEq/L), anemia, neutrophilia, and mildly elevated liver enzymes. Blood cultures subsequently grew *B. pseudomallei*. Lumbar puncture was performed. Cerebrospinal fluid (CSF) analysis demonstrated neutrophilic pleocytosis (cell count: 120 cells/mm³, predominantly neutrophils), elevated protein (160 mg/dL), and low glucose (35 mg/dL). CSF Gram stain showed no organisms, and CSF culture did not yield growth. Central nervous system (CNS) involvement is, therefore, classified as probable neuromelioidosis, based on consistent neuroimaging and blood-culture confirmation in the absence of CSF isolation of the organism [8].

**Figure 1:**
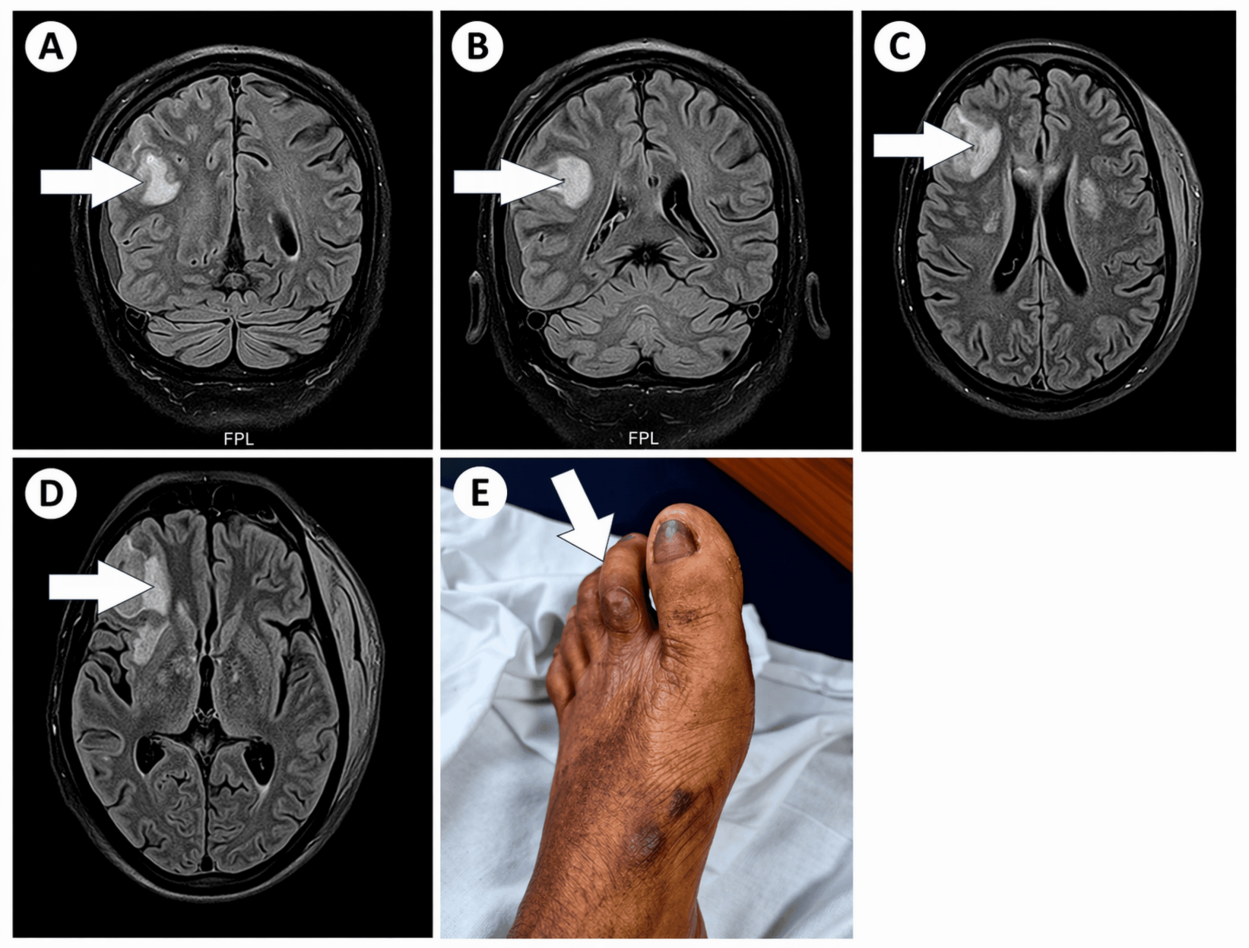
Composite imaging and clinical findings from three cases of melioidosis. A-C (Case 2): Axial and coronal fluid-attenuated inversion recovery (FLAIR) MRI images demonstrating a right frontoparietal cortical-subcortical hyperintense lesion with surrounding vasogenic edema (white arrows). An interhemispheric subdural and epidural collection and a 3.1 × 3.1 cm enhancing cortical lesion are evident. Although these features closely resemble tuberculous meningoencephalitis or pyogenic abscess, the imaging pattern in this case is attributed to probable neuromelioidosis based on: (1) blood culture confirmation of *B. pseudomallei*; (2) absence of basal meningeal enhancement or hydrocephalus, findings more characteristic of tuberculous meningitis; and (3) the subacute onset with diabetes as a risk factor. This distinction is clinically important in high-tuberculosis-burden settings [11,12]. D (Case 3): Axial FLAIR MRI image showing extensive right frontal cortical and subcortical hyperintensities with surrounding edema (white arrow), consistent with meningoencephalitic involvement. The imaging appearance mimics tuberculous meningitis or pyogenic abscess; however, neuromelioidosis is favored given blood culture-confirmed *B. pseudomallei* bacteremia, neutrophilic cerebrospinal fluid pleocytosis with markedly low glucose, absence of basal enhancement, and agricultural environmental exposure. This pattern has been described in prior central nervous system melioidosis reports. E (Case 3): Clinical photograph of the left foot demonstrating a traumatic lesion over the second toe (white arrow), representing a probable portal of entry for *B. pseudomallei* through environmental inoculation during barefoot agricultural work.

Neurological melioidosis, although uncommon, is recognized as a severe form of the disease [8], and experimental studies have demonstrated that the pathogen can invade the CNS through olfactory or trigeminal pathways [9,10]. MRI of the brain showed right frontoparietal cortical and subcortical hyperintensities, interhemispheric subdural and epidural fluid collections, and a 3.1 × 3.1 cm enhancing cortical lesion with surrounding edema (Figure 1, Panels A-C).

These findings closely resembled tuberculous or pyogenic meningoencephalitis, patterns well documented in prior neuromelioidosis case reports [11,12]. Neurosurgical biopsy was recommended for diagnostic confirmation, but it was declined by family members. No other neurosurgical intervention was pursued, given the absence of mass effect warranting surgical decompression. The planned total treatment duration was four to six weeks of IV meropenem followed by six months of oral TMP-SMX eradication therapy. He was treated with IV meropenem and dual antiepileptics; however, after 14 days of therapy, he was discharged against medical advice due to financial constraints. Incomplete antimicrobial therapy substantially increases the risk of relapse and mortality in CNS melioidosis [13], a risk that was clearly communicated to the patient and family at discharge. Limited follow-up was possible after discharge against medical advice; long-term outcome could not be established.

Case 3: Fulminant meningoencephalitis secondary to probable neuromelioidosis

A 62-year-old dairy farmer presented with a 15-day history of fever, progressively worsening headache, and altered mental status. Two weeks prior, he sustained a traumatic injury to his toe while working barefoot in wet soil, an exposure strongly associated with melioidosis in Indian agricultural settings [14]. On examination, he was febrile, disoriented, and had marked neck rigidity. Laboratory testing revealed hyponatremia (Na⁺: 126 mEq/L), hypoalbuminemia, elevated CRP, and neutrophilic leukocytosis. Lumbar puncture was performed. CSF analysis demonstrated neutrophilic pleocytosis (cell count: 150 cells/mm³), markedly elevated protein (180 mg/dL), and low glucose (30 mg/dL), consistent with bacterial meningitis. CSF Gram stain revealed no organisms, and CSF culture was sterile. CNS involvement is, therefore, classified as probable neuromelioidosis, supported by the CSF profile, neuroimaging findings, and blood culture confirmation. Two sets of blood cultures grew *B. pseudomallei*. MRI of the brain revealed extensive right frontal cortical and subcortical hyperintensities with surrounding edema, radiologic findings that closely mimic tuberculous meningitis or pyogenic abscesses, similar to previously described CNS melioidosis cases [15] (Figure 1, Panels D, E). Despite initiation of high-dose meropenem and aggressive supportive therapy, the patient’s neurological status deteriorated rapidly. He required mechanical ventilation and was ultimately discharged against medical advice while intubated. Long-term outcome could not be established due to discharge against medical advice while ventilated; no subsequent follow-up was available.

A consolidated overview of demographic characteristics, clinical features, laboratory findings, imaging, microbiological confirmation, treatment, and outcomes across all three cases is presented in Table 1.

**Table 1:** Consolidated overview of the clinical characteristics, laboratory findings, imaging results, microbiology, treatment course, and outcomes of all three patients. DAMA = discharged against medical advice; TMP-SMX = trimethoprim-sulfamethoxazole; HTN = hypertension; T2DM = type 2 diabetes mellitus; CRP = C-reactive protein; LFT = liver function test; CSF = cerebropinal fluid

Parameter	Case 1	Case 2	Case 3
Age, sex	58-year-old, male	60-year-old, male	62-year-old, male
Comorbidities	HTN, hepatitis B (past), T2DM	T2DM, HTN, chronic hepatitis B	None
Exposure	Raw/Unpasteurized milk	No known exposure	Dairy farmer; traumatic toe injury in wet soil
Presenting features	Fever, chills, gluteal swelling, loose stools	Fever, headache, seizures	Fever, headache, altered sensorium, neck rigidity
Key laboratory findings	Na⁺: 130 mEq/L, hemoglobin: 8.6 g/dL, platelet count: 80,000/µL, elevated CRP	Na⁺: 128 mEq/L, neutrophilia, elevated LFTs. CSF: 120 cells/mm³ (neutrophilic), protein: 160 mg/dL, glucose: 35 mg/dL, culture sterile	Na⁺ 126 mEq/L, elevated CRP, neutrophilic leukocytosis. CSF: 150 cells/mm³ (neutrophilic), protein: 180 mg/dL, glucose: 30 mg/dL, culture sterile
Imaging	Soft-tissue edema, splenomegaly, ascites	Subdural/Epidural collections; 3.1 cm cortical enhancing lesion	Extensive right frontal cortical/subcortical hyperintensities with oedema
Culture results	Blood culture: *B. pseudomallei* (×2)	Blood culture: *B. pseudomallei*; CSF culture: sterile	Blood culture: *B. pseudomallei* (×2); CSF culture: sterile
CNS designation	N/A (systemic)	Probable neuromelioidosis (blood-only isolation + neuroimaging)	Probable neuromelioidosis (blood-only isolation + CSF + neuroimaging)
Intensive therapy	IV meropenem 14 days (within Darwin guideline: 10–14 days)	IV meropenem initiated; planned 4–6 weeks; incomplete (14 days completed; DAMA)	Initiated; incomplete; deteriorated and DAMA while ventilated
Eradication therapy	Oral TMP-SMX 12 weeks (within Darwin guideline: 3–6 months)	Not completed	Not initiated
Outcome	Improved; stable at follow-up	Discharged DAMA after 14 days; limited follow-up possible; long-term outcome not established	Discharged DAMA while intubated; no subsequent follow-up available; long-term outcome unknown

## Discussion

Melioidosis remains a diagnostically challenging infection due to its ability to mimic a wide range of tropical diseases, particularly tuberculosis and pyogenic bacterial infections that are far more prevalent in India. The three cases presented here highlight the diverse clinical spectrum of melioidosis and emphasize the importance of maintaining a high index of suspicion, especially in patients from emerging endemic regions. To our knowledge, this is the first reported temporal cluster (three cases within three months) of culture-confirmed melioidosis from eastern India with a spectrum ranging from systemic bacteremia to fulminant CNS disease, a combination that has not been previously described from this geographic region, distinguishing this series from prior isolated case reports.

In Case 1, the presence of diabetes, a well-established risk factor for severe disease, likely contributed to systemic bacteremia, while in Cases 2 and 3, neurological involvement underscored the uncommon yet critical manifestations of neuromelioidosis. The imaging findings in these latter cases closely resembled tuberculous meningitis and pyogenic abscesses, a pattern frequently reported in the literature and one that often delays definitive diagnosis [11,12,15]. Environmental exposures also played an important role, with traumatic inoculation in Case 3 serving as a probable trigger, aligning with existing musculoskeletal and agricultural exposure data from India [14]. Experimental studies provide mechanistic insight into such CNS involvement, demonstrating that *B. pseudomallei* can gain access to the CNS via the olfactory and trigeminal neural pathways [9,10], explaining presentations where neurological signs dominate or precede systemic features.

Compared with prior Indian series, the imaging and clinical patterns in our cases align closely with those reported in the Tamil Nadu neuromelioidosis outbreak [6], the South Indian epidemiological series [7], and isolated CNS melioidosis case reports [11,12,15]. Corticospinal tract involvement and cortical hyperintensities on fluid-attenuated inversion recovery, as seen in Cases 2 and 3, mirror findings from Raj et al. [12] and Chatterjee et al. [11]. Notably, our cases share the same cardinal risk factors (diabetes, environmental inoculation) and the same radiologic mimicry of tuberculosis documented in the Darwin cohort [3]. What is distinctive about this series is the simultaneous occurrence of both systemic and neurological disease phenotypes at a single center in eastern India, a region previously underrepresented in melioidosis literature.

Laboratory findings of hyponatremia across all three cases reflect significant systemic inflammation and are consistent with previously reported patterns in severe melioidosis [8]. Blood cultures remained the definitive diagnostic modality in all cases, reinforcing their indispensable role in evaluating prolonged or unexplained febrile illnesses in endemic regions. In Cases 2 and 3, where CSF culture was sterile and *B. pseudomallei* was isolated only from blood, CNS involvement is classified as probable neuromelioidosis. CSF pleocytosis, elevated protein, and neuroimaging abnormalities serve as essential supportive diagnostic clues in blood culture-only confirmed cases [8,13].

Importantly, two of the three patients were unable to complete therapy due to socioeconomic constraints, a reality that poses substantial risks of relapse, treatment failure, and mortality, particularly in neurological disease, where prolonged eradication therapy is essential [13]. This is especially critical in CNS melioidosis, where incomplete intensive or eradication therapy is associated with significantly higher rates of relapse and death compared with systemic disease [13]. These observations highlight the urgent need for greater clinician awareness, early microbiological testing, enhanced imaging interpretation in high-tuberculosis-burden settings, and improved health system support to ensure treatment adherence. As melioidosis continues to emerge as a public health threat in India, addressing these diagnostic and therapeutic challenges is crucial to reducing morbidity and mortality associated with this under-recognized yet potentially fatal infection.

Limitations of this series include the small sample size (n = 3), the retrospective single-center design, the absence of CSF microbiological confirmation in the two neurological cases, the inability to complete therapy in two patients, and limited post-discharge follow-up. These constraints preclude generalizable conclusions, and findings should be interpreted within the context of this small, observational case series.

## Conclusions

Melioidosis should be strongly suspected in patients presenting with prolonged fever, soft-tissue lesions, or neurological deficits, particularly in individuals with diabetes or environmental exposure in emerging endemic regions. Radiologic findings frequently mimic tuberculosis or pyogenic abscesses, making microbiologic confirmation, primarily through blood cultures, essential. In the absence of CSF isolation of the organism, a diagnosis of probable neuromelioidosis can be supported by consistent neuroimaging, CSF pleocytosis, and clinical context. Adherence to prolonged intensive and eradication therapy is critical to prevent relapse. This is especially important in CNS-involved cases, where incomplete therapy substantially increases the risk of treatment failure and mortality. Socioeconomic challenges continue to limit treatment completion in India. Increased clinician awareness, improved diagnostic capacity, and treatment support programs are vital to reducing morbidity and mortality associated with this under-recognized infection.
